# Rheumatism, Rheumatic Gout, and Sciatica; Their Pathology, Symptoms, and Treatment

**Published:** 1855-01

**Authors:** 


ART. V.—*Rheumatism, Rheumatic Gout, and Sciatica; their Pathology, Symptoms, and Treatment.* By HENRY WILLIAM FULLER, M. D., Cantab., Assistant Physician to St. George's Hospital, &c., &c. 1 vol. 8mo., pp. 322. (From the Publishers, S. S. & W. Wood, New York.)

This is a very valuable treatise on the subjects above-mentioned. It is conceived in the true philosophical spirit, and also, considering all the circumstances connected with the investigation of Rheumatism, very well executed. We do not adopt all the conclusions at which the author arrives, without the aid of farther investigations. But it is at least an able effort in the right direction, and its *progressive* character entitles it to a thorough notice here.

The work is divided into thirteen chapters, upon the following topics:

I. In the *introductory* chapter the author objects to the theory so generally adopted that rheumatism is produced by cold, or by the combined influence of cold and moisture; and argues that it is probably due to the presence of a morbid matter in the blood—that this matter is a product of mal-assimilation, or vicious metamorphic action—that it is in fact probably *lactic acid*—and that at least the poison is of a *specific character.*

We cannot recapitulate the facts and reasons adduced in favor these propositions. We merely remark that in the present state of physiological and pathological science, we believe they cannot be gainsayed. We have ourselves for years adopted the idea, and taught that acute rheumatism is a *blood* disease, that the *materies morbi* is an *acid*, and probably the *lactic.*

The phrase "rheumatic virus," does not, therefore, take us by surprise, nor the announcement that "this poison it is which excites the fever, and produces all the pains and local inflammations, which are often found associated in an attack of rheumatism." From this idea of the proximate or essential cause of the disease, it must also follow that cold and other external agencies, can be nothing more than predisposing and exciting causes of the same, as the author maintains.

II. The second chapter treats of the *rheumatic diathesis* and the *causes which influence its development.*

Rheumatism, like gout, is *distinctly hereditary*. The author has traced its hereditary character in nearly twenty-nine per cent. of the rheumatic patients admitted into St. George's Hospital; and Chomel ascertained it in half the cases admitted into the Hotel Dieu. This character also almost invariably exists in the cases which are earliest in life, and most fully developed.

1st. The *age* at which the disease is most frequently developed is considered. Very few cases occur before the age of 15; and comparatively few after the age of 50. Of 246 cases cited by the author, there occurred

| | | |
|---|---|---|
| Under 15 years, | - - - | 15 cases. |
| 15 to 20 " | - - - - - | 58 " |
| 20 to 25 " | - - - - - | 63 " |
| 25 to 30 " | - - - - - | 50 " |
| 30 to 35 " | - - - - - | 22 " |
| 35 to 40 " | - - - - - | 17 " |
| 40 to 45 " | - - - - - | 9 " |
| 45 to 50 " | - - - - - | 9 " |
| 50 to 55 " | - - - - - | 1 " |
| 55 to 60 " | - - - - - | 2 " |

It will be noticed that the greatest number of cases occurred in the five years from 20 to 25; next from 15 to 20; and thirdly from 25 to 30. It will also appear that nearly one-half of all the cases (121) occurred during the decade from 15 years to 25. Chomel and McLeod have arrived at very similar results.

In 7 out of 73 of Chomel's patients, the disease occurred after 60 years of age; while others—Drs. Heberden, Watson, and the author—have seen it in children from 2 years and 9 months upward.

2d. The acute *form* of the disease is most common in early life; the chronic form in advanced life. An acute attack does *not*, however, as sometimes asserted, strengthen the disposition to rheumatism, though there is much reason to dread its recurrence.

3d. Those most exposed to the existing causes of the disease, (atmospheric vicissitudes, and mal-assimilation from bad diet, irregularity of life, &c.,) are, of course, the most obnoxious to rheumatism. Hence it is more common in the male sex, and in the lower classes.

4th. *Climate* and *season* affect the development of the disease; but indirectly, and as mere predisposing causes. As cold and damp diminish the activity of the skin through which much of the lactic acid accumulating in the blood is excreted; cold weather and the colder seasons, are most favorable for its development. From observations made in India by Dr. Malcolmson, it appears that the acute form at least of this disease, is very rare within the tropics. It is also known to be uncommon toward the poles. In the torrid zone the skin is constantly active; in the frigid the great bodily vigor and the simple diet of the natives, prevent mal-assimilation. Chomel also remarked that this disease prevails especially in the temperate zones.

We have not the space for the statistics which the author adduces in support of the preceding views.

III. The third chapter treats of the *seat* of rheumatism, and the *classification* of its varieties.

Being a *blood disease*, rheumatism should not be confined to any special seat. Hence we find it may produce its symptoms in very different tissues and organs. The joints and surrounding parts, are most frequently affected. But it also attacks the pericardium, the endocardium, and sometimes the heart itself; the uterus, the kidneys, the liver, and the lungs, may be affected by it; active pleurisy, and even peritonitis, may thus ensue; the eyes occasionally suffer, as do also the tastes and the skin, and the periosteum in different parts of the body. Rheumatic inflammation of the membrane of the spinal cord, is also admitted by Dr. Copland; and a similar condition of the dura-mater is spoken of by most writers on this disease.

Still the rheumatic poison, like other poisons, manifests a preference for a particular tissue. This is the *white fibrous tissue*, and the latter is the predominating element in the structure of the joints, the periosteum, the dura-mater, and the valves of the heart.

No perfectly conclusive explanation of these facts can be given. It is still interesting to remark, however, that the above are examples of the albuminous and gelatinous tissues, from the metamorphosis of which in the body result "lithic, (uric,) and lactic acids, with which gout and rheumatism are intimately connected." And the author adds, that "some particular attraction may be exerted by the fibrous and fibro-serous textures for compounds, such as the lithic and lactic acids, to which they bear so strong an affinity."

We would suggest, on the other hand, that these acids are not *attracted by* the tissues at all. But an accumulation of the acids in the blood, from whatever cause, must at once modify the nutrition of these tissues, since it will prevent the entrance into the already surcharged blood of these acids resulting from their metamorphosis, (wear and tear,) in the living body. Thus the consequence is a retention or *deposit* of these acids for the time in these tissues; this arrests the nutritive changes in them, the circulation in their capilliaries becomes modified, and the inflammation constituting the disease, is the result. Hence if we *remove the acid from the blood*—whether by alkalies, by increasing the action of the skin, or through the alimentary canal or the kidneys—we thus at length restore the circulation and nutrition of the part affected, and remove the disease. And hence the success obtained by such different and apparently opposite methods of treating this disease. But we return to our task.

It is well known that the joints most exposed, (*e. g.* the knees,) are the most likely to suffer. Moreover those already weakened by previous injury, or a previous attack of this disease, are very liable to become affected.*

The author admits there is no propriety, or pathological grounds, in admitting *varieties* of rheumatism. For *practical* purposes, however, he admits the following modifications:

1st. Acute rheumatism, or rheumatic fever.

2d. Rheumatic gout.

3d. Chronic rheumatism.

4th. Neuralgic rheumatism.

Chapters 4th to 10th, inclusive, are devoted to the consideration of Acute Rheumatism; chapter 11th to Rheumatic Gout; the 12th chapter to Chronic Rheumatism; and the 13th and last, to Sciatica, and other forms of Neuralgic Rheumatism.

### *Acute Rheumatism.*

IV. In the fourth chapter the author asserts the extreme danger of this disease, from its tendency to invade the heart; speaks of its various modes of attack, and of its symptoms, local and general; and shows that the febrile symptoms are not commensurate with the extent and intensity of the articular inflammation; and that the perspiration often spoken of as "enfeebling and useless," is *not* so, in fact. He states the symptoms indicative of the subsidence of the disease; guards against mistaking the stiffness left after an acute attack, for the chronic form of the disease; and introduces statistics to show that its ordinary duration is thirty-five days. He believes that the paroxysm cannot be *cut short* or *arrested* by treatment, and explains away the cases cited in proof of the opposite opinion.

V. In the fifth chapter we find the *treatment* of acute rheumatism.

---

* We explain this by the suggestion already made. The circulation already debilitated in the part, more easily yields to a farther disturbance from the cause before specified, than it does in other parts not thus predisposed. E. R. P.

The author specifies the various remedies employed in the hitherto uncertain and contradictory treatment of this disease; assigns the cause of this uncertainty, the grounds on which the rational treatment should be based, the objects to be effected, and the means of fulfilling them.

He then considers the value of different remedies—bleeding, purging, opium, vapor and hot-air baths, mercury, tartar-emetic, cinchona, colchicum, guaiacum, nitrate of potash, lemon juice, and alkalies and their salts.

We regard this part of the work as very discriminating and judicious, and especially commend it to the careful study of practitioners of medicine. He thinks that particular remedies, or classes of remedies, are generally relied upon too exclusively in the treatment of this disease; and concludes the chapter with an account of his own treatment, and statistics showing its results: "It is made up of alkalies, with the neutral salts, with colchicum, calomel and opium. Sometimes a little antimony is added, sometimes the aid of purgatives is had recourse to, and occasionally, though rarely, I deem it expedient to premise a moderate bloodletting. Baths are never employed if the skin is acting freely; but if instead of being bathed in perspiration, it remains dry and hot, and burning, I then endeavor to stimulate its action by means of the vapor or the hot-air bath."—p. 96. The influence of the author's treatment over the pulse and the urinary secretions, and in subduing the pain and arresting the progress of the inflammation, is shown to be very marked, by illustrative cases.

VI. The sixth, seventh, eighth, and ninth chapters, are upon *rheumatic inflammation of the heart.*

In chapter fifth the author considers the *causes* of rheumatic affection of the heart, which is now recognized as imparting to rheumatism its chief danger.

VII. Chapter seventh includes the *pathological effects*—the *symptoms, progress,* and *terminations* of this affection. The pericardium once inflamed, is rarely restored to a healthy state. A complete recovery after an attack of endocarditis is possible, but it is extremely improbable, when fibrinous deposits have taken place on the valves.

The symptoms of pericarditis, endocarditis, and rheumatic carditis, are minutely detailed; and head symptoms occurring in the course of acute rheumatism, are shown usually to be indicative of inflammation of the heart, (carditis.)

VIII. The eighth chapter discusses the *treatment* of rheumatic affection of the heart.

IX. The ninth chapter gives the *statistics of the heart* disease, in connection with acute rheumatism.

It appears that 306.5 cases of recent heart affection occurred out of 588 cases of acute rheumatism, collected by several writers: or 1 in every 1.91, or *slightly above one-half of all the cases. Pericarditis* occurred in 1 out of 6.3 of the author's cases; and *endocarditis* in 1 out of 2.3 cases. Putting together these two affections, they constitute 1 out of ·2.7 of all the author's cases.

We regret that we cannot do justice to the last mentioned chapters. The portion of the work devoted to affections of the heart, will be considered proportionably too great by those only who are unacquainted with the great practical importance of this subject.

X. In the tenth chapter the author considers *affections of the brain, inflammation of the lungs and pleuræ, and disorganization of the joints*, as complications and consequences of acute rheumatism. Delirium occurring, is rarely (though sometimes) referable to inflammation of the brain; but is generally connected with inflammation of some internal organ. Convulsive and spasmodic symptoms, indicating spinal irritation, are also due to the same circumstances. Cerebro-spinal symptoms are always indicative of great danger; but not necessarily of a fatal termination of the disease. The author adduces cases in demonstration of these propositions, and adds some practical suggestions respecting the treatment of cases in which cerebro-spinal symptoms present themselves.

Inflammation of the lungs and pleuræ, is a very formidable complication of acute rheumatism; and *double* bronchitis, pneumonitis, or pleurisy, is very common. Dr. Latham reports some form of pulmonary complication in 1 out of every 5.6 cases of acute rheumatism; and the author found exactly 1 in 6 cases. The symptoms are severe, as the whole blood is poisoned; and great caution in the use of depleting measures is necessary.

Another formidable complication is *disorganization of the joints.* Generally this is more liable to occur in proportion as the number of joints affected is *less.* Particular attention must be paid to the earliest signs of commencing mischief; and the author especially recommends the application to the affected part of an alkaline and sedative solution, as follows:

℞ Potassæ carb., ℥j.
Liquor opii sedativ., ℥vi.
Aquæ rosarum, ℥ix.

Apply a thin flannel over the joint, soaked in the above, and then a covering of oiled silk.

We can only glance at the contents of the three remaining chapters.

XI. Chapter eleventh on *rheumatic gout*, gives an account of its symptoms, the distortions it produces, and the treatment to be adopted. The *eye* is often affected in rheumatic gout, (about 1 in 15 cases,) and but rarely in acute rheumatism.

XII. The twelfth chapter is on *chronic rheumatism*, which is identical with the acute in its source and nature, and may light up into the latter, as the latter may lapse into the chronic form. Of the various *forms*, the author speaks of muscular rheumatism, (often leading to the wasting of the limbs); lumbago, which, if neglected, is liable to become very obstinate; wry-neck; intercostal rheumatism, and periosteal rheumatism. The pains of chronic rheumatism are most acute at night, and are sometimes aggravated by heat.

The iodide of potassium is most useful in periosteal rheumatism, and the hydrochlorate of ammonia in muscular rheumatism: the former doses of two or three grains, and the latter of fifteen to twenty grains, with bark. Warm applications are of great value in lumbago and wry-neck. The application of sulph. ether or chloroform, also, sometimes affords great relief. But this chapter demands a very careful perusal.

XIII. The last chapter treats of *sciatica, and other forms of neuralgic rheumatism.* This is also a chapter of great practical value. It shows the relation of sciatica and other kinds of neuralgic rheumatism to the other forms of rheumatism before-mentioned, and how to distinguish them; and discusses the value of opium, belladonna, stramonium, hyorcyamus, and conium as sedatives; and of quinia, iron, and arsenic as tonics. We have long *felt* the relationship between sciatica and acute rheumatism; we have never clearly understood it till we perused this chapter.

We close by unqualifiedly recommending this book to the profession, not as free from imperfections, by any means, but as a work decidedly in advance of any other monograph upon the different forms of rheumatism, up to the present time. E. R. P.

